# Can Mobile Payment Increase Household Income and Mitigate the Lower Income Condition Caused by Health Risks? Evidence from Rural China

**DOI:** 10.3390/ijerph191811739

**Published:** 2022-09-17

**Authors:** Weisong Qiu, Tieqi Wu, Peng Xue

**Affiliations:** 1 Tailong Finance School, Zhejiang Gongshang University, Hangzhou 310018, China; 2 The School of Management and Economics, Jingdezhen Ceramic University, Jingdezhen 333403, China; 3 The Six Topographic Survey Team of Ministry of Natural Resources, Chengdu 610500, China

**Keywords:** mobile payment, household income, health risks, rural China, poverty alleviation

## Abstract

China has moved into a new stage of its fight against poverty, where the further raising of rural household income is of great importance. Health risk is one of the biggest obstacles to the poverty reduction progress. Therefore, how to cope with the negative effects of health risks has attracted the attention of scholars, especially in the background of the global outbreak of COVID-19. In this paper, we try to explore whether mobile payment, a new form of payment, can improve the income of rural households and mitigate the lower income condition caused by health risks in China. Using data from the 2017 China Household Finance Survey, we found: (1) mobile payment can substantially increase rural household income; (2) health risks will lower the income of rural residents, but mobile payment can lessen this negative effect. Mechanism analysis indicates that mobile payment is likely to ease liquidity constraints, increase social interaction, and stimulate entrepreneurship for rural households. We advised the government to promote mobile payment adoption in rural areas and enhance its design. Additionally, better medical resources should also be made available to rural households.

## 1. Introduction

As the largest developing country, China has achieved considerable achievement in the alleviation of poverty. By the end of 2020, 98.99 million rural residents have been lifted out of absolute poverty [1]. Now, China proposes new goals regarding poverty alleviation, transitioning from solving absolute poverty to alleviating relative poverty and from phased poverty reduction to sustainable poverty reduction [2]. Therefore, it is of great importance to continue to improve the income of rural households in China [3]. Health risk is one of the biggest obstacles to the progress of poverty reduction. Compared with urban households, the health condition of rural households is worse and their access to medical resources is poorer [4,5]. Health risks have adverse consequences for rural households and can push them into poorness [6,7,8,9,10].

The ongoing COVID-19 pandemic has created a further challenge for poverty alleviation. Since January 2020, COVID-19 has spread rapidly and has been declared a major public health emergency globally. As of 30 August 2022, the pandemic had infected 596,873,121 people and killed 6,459,684 people worldwide, according to the World Health Organization. The sudden outbreak affected a wide range of regions and lasted for a long time, having far-reaching economic and environmental consequences for countries all over the world [11,12,13,14,15]. On the one hand, COVID-19 is associated with air quality improvements and less environmental noise because of declining economic activities [16,17]. On the other hand, lockdowns and economic disruption caused by the outbreak have resulted in a sharp drop in household income in both developed and developing nations [18,19], pushing millions of people into extreme poverty [20]. The situation for rural households is even worse [21]. Studies have shown that they are more negatively affected by the COVID-19 pandemic [22,23]. As a result, the government is concerned with how to mitigate the impact of the COVID-19 pandemic, not only on economic growth but also on the well-being of rural groups.

Because of the rapid development of information and communication technology, mobile payment in China has grown explosively since 2013 [24]. Mobile payment meets the demand for convenience of modern people and significantly alters our daily lives by providing widespread access, low transaction costs, and secure transactions [25,26]. During the COVID-19 pandemic, mobile payment usage increased significantly because it can reduce contact and maintain social distance between people [27,28]. The growing popularity of mobile payments has inevitably drawn the attention of academics to their effects on households.

In this paper, we try to explore whether mobile payment can improve the income of rural households and mitigate the lower income condition caused by health risks in China. Based on data from the China Household Finance Survey (CHFS) in 2017, we conducted several empirical tests and reached rewarding conclusions. First, we used the OLS model to run the regressions and found that mobile payment can substantially improve rural household income, measured by the annual total income and per capita income. Second, we utilized the IV method and the PSM method to solve the endogeneity problems and self-selection problems of the OLS model. Our results are consistent with baseline regression. Third, we explored the moderating effect of mobile payment. Results show that health risks will lower rural residents’ income, but mobile payment can mitigate this negative effect. Fourth, we investigated three possible channels through which mobile payment functions. Our empirical results show that mobile payment is likely to ease liquidity constraints, increase social interaction, and stimulate entrepreneurship for rural households.

This paper contributes to the growing body of research in several ways. First, this paper is among the first, to the best of our knowledge, to explore the role of mobile payment on rural household income and its moderating effect on health risks in China, which adds to the literature on mobile payment. On the one hand, most research about mobile payment is restricted to just a few African countries [29,30,31,32]. Further empirical evidences from other developing countries are urgently needed. On the other hand, this study tests whether mobile payment can lessen the negative impact of health risks on rural residents, which has not been covered in the existing literature. Second, our results have important policy implications. For one thing, China leads mobile payment markets in terms of both the number of active users and the volume of transactions currently [24]. Our study has important policy implications, not only for China, but also for other countries inclined to reduce poverty or develop mobile payments, especially in developing countries. Furthermore, our findings have important implications for dealing with health risks, shedding light on how to manage the negative effects of COVID-19.

The remainder of this paper is as follows. Section 2 introduces background information, literature review, and theoretical analysis. Section 3 discusses our data and empirical strategy. Section 4 presents the empirical results. Section 5 provides a brief discussion. Section 6 concludes.

## 2. Background, Literature Review, and Theoretical Analysis

### 2.1. Background

**Figure 1:**
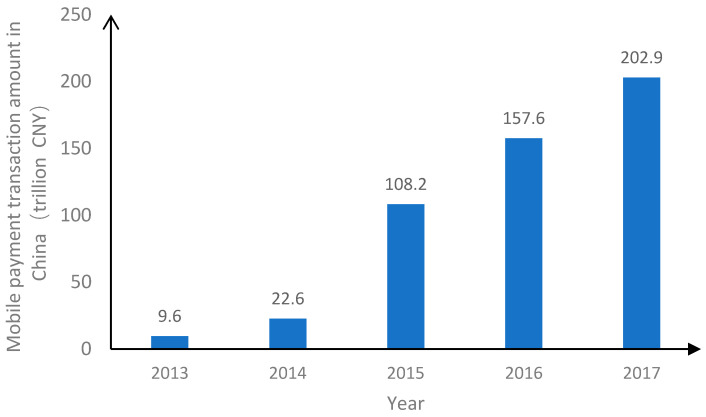
The market transaction volume of China mobile payment (Data source: The People’s Bank of China).

**Figure 2:**
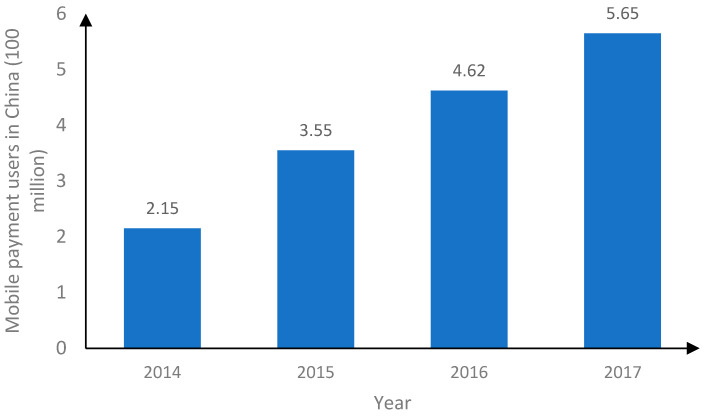
The number of users of China mobile payment (Data source: The People’s Bank of China).

In China, the concept of mobile payment was proposed in 1999, but it was not promoted because of the lagging information and communication technology at that time. Since 2011, the development of financial technology and mobile Internet technology has grown rapidly. At the same time, the People’s Bank of China granted a third-party payment license to payment platforms (e.g., UnionPay, UnionPay Commerce, Alipay, and TenPay), laying the groundwork for the development of mobile payment. According to statistics from the People’s Bank of China (Figure 1 and Figure 2), the market transaction volume of China’s mobile payment was only CNY 9.6 trillion in 2013 and exceeded CNY 200 trillion in 2017. Meanwhile, the number of users increased from 215 million in 2014 to 565 million in 2017.

For now, Alipay and WeChat Pay dominate the mobile payment market. With the installation of Alipay or WeChat Pay apps on smartphones, iPads, or other mobile devices, people can transfer money or pay fees through mobile payment instantly, securely, and cheaply. Mobile payment has become a global phenomenon, which sparks intense curiosity among academics, business professionals, and policymakers. Can it be done in other nations as well? What are the main implications? How ought it to be governed? These questions are being addressed in a growing body of literature.

### 2.2. Literature Review

Our study is related to two streams of the literature. The first one is the studies on the impact of mobile payment on households. Mobile payment can increase the welfare of households. Aker et al. (2016) showed that the introduction of mobile payment could broaden the diet diversity of Niger households [29]. Based on survey data collected in Kenya, Kikulwe et al. (2014) found that mobile payment has a positive impact on household income [33]. Using the same data, Suri and Jack (2016) discovered that the usage of mobile payment can lift households out of poverty and increase their consumption [31]. China has also seen the benefits of mobile payment for households. According to Zhao et al. (2022), vulnerable populations will benefit more from mobile payment’s favorable effects on consumption [34]. By exploring the data from the China Household Finance Survey, Zhao et al. (2022) revealed that the subjective well-being of mobile payment users is higher than that of non-users [35].

The second one is the studies on the health risks. Health risks can cause significant adverse economic outcomes for households, which are more pronounced for rural residents [36]. Health risks may cause rural households to return to poverty again and are an important determinant of long-term poverty [37]. As a result, it is important to help rural households insure against health risks. By utilizing a cross-sectional survey among 1226 agricultural households in Kenya, Bonfrer and Gustafsson-Wright (2017) identified three main coping strategies (using savings, selling assets, and asking for gifts or loans) when rural residents face health risks [38]. This finding is also supported by Mitra et al. (2016) [39] based on data from Vietnam and Islam and Maitra (2012) [8] based on data from Bangladesh. Health insurance is another way to manage health risks in developing countries [10,40,41]. In addition, village elections can also reduce the negative effects of health risks, according to Zhang et al. (2014), who used a sample of 1185 families from 48 Chinese villages [7]. 

### 2.3. Theoretical Analysis

We identified three possible mechanisms through which mobile payment might operate. This includes easing liquidity constraints, increasing social interaction, and stimulating entrepreneurship.

The first possible channel is easing liquidity constraints. Financial support can effectively reduce poverty and the harmful impacts of health risks [8,42]. However, the issues of high-risk exposure, high fixed growth costs, and low investment returns are frequently linked to the development of financial inclusion in rural areas [43,44]. As a result, traditional financial institutions are reluctant to provide financial services to rural households [45,46]. Mobile payment can ease liquidity constraints in the following way. First, small loan services based on mobile payment have been made available by WeChat Pay and Alipay. These loans have a lower qualification requirement and an easier application process than bank loans [47]. In addition, by utilizing big data-based risk evaluation models through mobile payment, traditional financial institutions could effectively reduce the degree of information asymmetry when providing financial services [48].

Social interaction is the second potential pathway. In rural China, social networks have long been considered to have a significant impact on households’ economic behavior and daily life. On the one hand, studies have shown social interaction, as an important human capital, can boost the income level of rural residents [49]. On the other hand, for developing countries, such as China, where insurance markets are underdeveloped, social interaction is an important means of risk sharing [50]. Mobile payment can bring more frequent online or offline communication and social contact between households [35,51], which increases social interaction. For example, WeChat Pay is a product of China’s largest online social platform, WeChat. WeChat users can communicate, engage in online entertainment, transfer money, make reservations for restaurants or lodging, among other things.

The last possible channel is stimulating entrepreneurship. It has been demonstrated that entrepreneurship can increase the income and concentrate the wealth of households [52,53,54]. Mobile payment makes entrepreneurship more possible. For one thing, mobile payment can effectively improve access to financial services for entrepreneurs as we mentioned above. Furthermore, mobile payment can provide entrepreneurs with an effective channel to explore and grasp more business opportunities and information [51,55]. 

**Figure 3:**
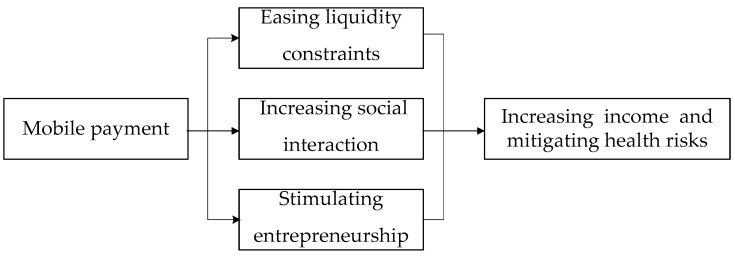
Theoretical analysis framework.

In conclusion, the theoretical analysis framework of this paper is shown in Figure 3. The following background information led us to our hypotheses:

**Hypothesis** **1.** *Mobile payment can increase rural household income.*

**Hypothesis** **2.** *Health risks have a negative impact on rural households’ income, but mobile payment can mitigate this negative impact.*

## 3. Research Design

### 3.1. Data

We used the data from the China Household Finance Survey (CHFS) provided by the Southwestern University of Finance and Economics to conduct our research. With the aim of collecting exhaustive information on household finance, CHFS has been performed every two years since 2011, and was subsequently conducted in 2013, 2015, 2017, and 2019. The survey includes information about assets and liabilities, income and expenditures, social security and insurance, demographic characteristics, employment status, and many others, providing a comprehensive and detailed description of household economic and financial behaviors. 

Among the five rounds of CHFS, only the fourth round of the survey (CHFS 2017) asked about payment methods that households use when shopping. Therefore, we use data from CHFS 2017 in this study. The 2017 survey covered 29 provinces (excluding Xinjiang, Tibet, Hong Kong, Macao, and Taiwan), 355 cities and counties, and 1439 communities and villages, obtaining a valid household sample of 40,011 and a valid individual sample of 127,012.

### 3.2. Methods

The baseline specification for estimating the effect of mobile payment on rural household income is

(1) $$y_{i}=\alpha +\beta mobile\_payment_{i}+\gamma X_{i}+c_{j}+\varepsilon_{i}$$

Equation (1) is the ordinary least squares (OLS) model, where $y_{i}$ is the outcome variable: the income of household i. The variable of interest, mobile_payment, is a dummy variable and β is the coefficient of interest to be estimated. *X* is a vector of covariates that includes demographic, household, and regional characteristics and γ is the coefficients of them. In addition, α is the constant term, $c_{j}$ is the province fixed effect, and $\varepsilon_{i}$ is the error term.

One concern with our model is the potential endogeneity issue. First, high-income households are more likely to use mobile payment. In addition, unobserved factors such as cultural factors or personality traits may influence mobile payment usage. Therefore, we used the Instrumental Variable (IV) method to solve the endogeneity problem. We utilized the mobile payment usage rate of the other households within a village/community as an instrumental variable. The mobile payment usage of an individual within a village/community can be influenced by other people and is unlikely to affect household income through the other channels [34,56].

Another concern is the self-selection bias [57]. Households that use mobile payment are naturally self-selected. Therefore, we use the Propensity Score Matching (PSM) method to examine the self-selection problem. Households who use mobile payment were determined as the treatment group, and those who do not were treated as the control group. By matching members with similar individual features between the treatment group and the control group, we can obtain the Average Treatment Effects (ATT) on Treated. The specific calculation formula of ATT is:

(2) $$\;\;\;\;\;\;\;\;\;\;\;\;\;\;\;\;\mathrm{ATT}=E\left[y_{1i}\left|mobile\_payment_{i}=1]-E[y_{0i}\right|mobile\_payment_{i}=0\right]$$

where $y_{1i}$ represents the income of rural households who use mobile payment, and y_0*i*_ represents the income of households who do not use mobile payment, which is constructed through the counterfactual framework.

To test whether health risks will lower the income level of rural residents, we applied the OLS model to Equation (3):

(3) $$y_{i}=\alpha +\beta health\_risk_{i}+\gamma X_{i}+c_{j}+\varepsilon_{i}$$

where health_risk is our focus variable and the other variables are the same as Equation (1). Then, following Yin et al. (2019) [51] and He et al. (2022) [58], we divided samples in two groups depending on whether they had unhealthy household members. The effects of mobile payment on rural household income will be examined in each group to verify its moderating role.

### 3.3. Variables

We used household annual total income and per capita income to evaluate household income. We obtained the per capita income by dividing the household’s total income by the household size. The logarithmic form of household income and per capita income is used in regressions.

The main explanatory variable in this study is the use of mobile payment. There are five payment method options in the questionnaire: cash, card, computer, mobile payment, and others. Based on each householder’s response to this question, we have defined the explanatory variable, mobile payment, that equals one if the rural household uses mobile terminal payment when shopping, and zero otherwise. Another explanatory variable we focus on is the health risks of the household. According to Wang et al. (2021), we use the number of unhealthy family members to measure it [59]. 

Other control variables include demographic, household, and regional characteristics. Demographic characteristics consist of age, gender, education, marital status, and employment status. Household characteristics are household asset, the number of household members, the number of labor force, and entrepreneurship. Regional characteristics include GDP per capita. The definitions of all variables are in Table 1.

### 3.4. Descriptive Statistics

Prior to the analysis, we kept samples from rural areas and excluded samples with the following characteristics: (1) head of households under the age of 16; (2) households with missing variables; (3) households with assets less than or equal to zero. Finally, we constructed a cross-section dataset with 12,318 observations to study the causal relationship between mobile payment and household income. Table 2 and Table 3 report the summary statistics of the key variables.

As seen in Table 2, only 11% of rural households use mobile payment when shopping. Table 3 reports the results of the mean difference in income between rural households with and without mobile payment. Rural households who use mobile payment typically have higher income levels.

## 4. Results

### 4.1. Baseline Analysis

First, we used OLS models to analyze the relationship between mobile payment and rural household income, measured by the annual total income and per capita income. The regression results are reported in Table 4. In columns (1) and (3), only the usage of mobile payment is added into the estimations. The results show that the income level of rural households is significantly higher when they use mobile payment. In columns (2) and (4), all control variables are included in the regressions, and the coefficients of mobile payment are still significantly positive at the 1% level. After considering the effect of other control variables, the total income and per capita income of rural households who use mobile payment are 30.6% and 30.5% higher than those who do not. Table 4 demonstrates that the usage of mobile payment can increase rural household income, which supports Hypothesis 1.

Among other control variables, the income of married rural households is higher than that of unmarried rural households. Along with the level of education, rural household income also grows significantly. These echo the views that marriage and education are important human capital [60,61]. The employment situation has an impact on household income as well, with employed households earning significantly more. As an indicator of economic development, GDP per capita is significantly positive, indicating households living in wealthier regions have a higher income.

### 4.2. Robustness Check

To overcome the endogeneity between household income and mobile payment, we applied the IV method for further estimation. Specifically, the mobile payment usage rate of the other households within a village/community is used as the IV. We assumed that mobile payment usage in the region would influence individual households’ decisions to use mobile payment, but not their income, so the IV is exogenous. 

Table 5 reports the result of instrumental variable estimation. The F values in the first stage are 40.51, and the P values of the Durbin–Wu–Hausman test are all less than 0.01, indicating that the IV meets the correlation restriction and does not have a weak instrument variable problem. The second stage estimation results show that the usage of mobile payment is still significantly positive at 1%, implying a positive relationship between mobile payment adoption and rural household income.

The self-selection bias in the OLS estimation is another problem. We used the propensity score matching method to examine the self-selection problem. The treatment group consists of households that use mobile payment as specified in Section 3, while the control group consists of households who do not. We applied a variety of matching techniques, including nearest neighbor matching (*k* = 1), nearest neighbor matching (*k* = 4), and radius matching (*r* = 0.01), to strengthen and increase the reliability of the results. The ATT outcomes of the various matching methods are presented in Table 6. 

Although the computed outcomes of various matching techniques vary slightly, their general trends are the same. The income level of rural households who use mobile payment is significantly higher than those who do not, which is consistent with the benchmark regression results.

### 4.3. Mitigated Effects of Mobile Payment on Households with Health Risks 

In this part, we first explored how health risks affect rural household income. Table 7 displays the outcomes of Equation (3). Coefficients of the number of unhealthy household members are significantly negative at the 1% level, indicating that the income level in households with higher health risks is relatively lower. In other words, health risks will lower rural household income. This is consistent with Wang et al. (2021) [59]. Farmers will experience lower agricultural production efficiency and higher medical costs as a result of health risks, resulting in a decrease in household income.

Then, we further investigated the mitigated effects of mobile payment on health risks. 

As we mentioned in Section 3, we divided households into two groups based on whether they had unhealthy household members and tested the effects of mobile payment in each group according to Yin et al. (2019) [51] and He et al. (2022) [58]. The results are reported in Table 8. The coefficient of mobile payment in households with health risks is higher than in those without, implying that mobile payment has a bigger impact on the income of rural households with health risks. These results suggest that usage of mobile payment can mitigate the negative impact of health risks on rural household income, which is consistent with the view that the positive effects of mobile payments are more pronounced among disadvantaged groups [31,34]. Table 7 and Table 8 support Hypothesis 2.

### 4.4. Mechanism Analysis

In this subsection, we investigated how mobile payment improves the income of rural households and mitigates the lower income condition caused by health risks. We tested the possible mechanisms proposed in Section 2. As noted earlier, mobile payment can make it easier for rural households to obtain loans from mobile payment platforms and traditional financial institutions. However, due to data availability, we are unable to observe whether rural households have made small loans through mobile payment, but can identify the loans made by traditional financial institutions. Therefore, we selected two binary variables, Bank Loan and Credit Card, to measure the households’ access to financial resources. Bank Loan (Credit Card) equals one if the household has bank loans (credit cards). As shown in Table 9, mobile payment increases the potential of households to acquire bank loans and credit cards at the 1% level. In summary, mobile payment helps rural households obtain financial support.

Next, we considered whether mobile payment can increase social interaction. Following Liang and Guo (2015), we used transfer expenditure and communication costs as proxy variables of social networks [62]. Transfer expenditure means a household’s expenditures on cash or gifts to non-family members for (1) festivals; (2) weddings and funerals (including birthday gifts); (3) healthcare and education. The communication costs include the fees for telephone and Internet access. We used the logarithmic form of these two variables to conduct regression analysis. The coefficients of mobile payment are significantly positive in Table 10, which means rural households that use mobile payment are more active in social interaction than those who do not. Mobile payment can broaden households’ social network.

Finally, we explored the impact of mobile payment on entrepreneurship. In CHFS, there are two types of entrepreneurships: formal and informal. Formal entrepreneurship covers four different types: sole proprietorships, partnerships, limited liability companies, and businesses limited by shares. Unregistered companies and registered self-employed businesses are both examples of informal entrepreneurship. In Table 11, the estimates from the OLS models suggest that mobile payment usage significantly increases the likelihood of entrepreneurship.

Based on the above analysis, we conclude that mobile payment functions by easing liquidity constraints, increasing social interaction, and stimulating entrepreneurship.

## 5. Discussion

The new goal of poverty reduction urges the Chinese government to continue increasing the income of rural households. Health risk is one of the biggest obstacles to the poverty reduction progress. Therefore, how to cope with the negative effects of health risks has attracted the attention of scholars, especially in the background of the global outbreak of COVID-19. Based on data from CHFS 2017, we used the OLS method to explore whether mobile payment can improve the income of rural households and mitigate the lower income condition caused by health risks in China. We also examined potential channels through which mobile payments might perform. The research results have strong practical significance for China and other developing countries.

We found that mobile payment can substantially improve the income of rural households. This is consistent with the results of Suri and Jack (2016) [31] and Kikulwe et al. (2014) [33]. They both found that the income of households who use mobile payment is higher than those who do not, in Kenya. We also found that health risks will lower rural household income, which is in accordance with previous research [4,5,6,7,59]. In order to better reflect the differences in research areas, this study found that mobile payment can lessen the negative effect of health risks. Mechanism analysis shows that mobile payment can ease liquidity constraints, increase social interaction, and stimulate entrepreneurship, which helps us understand the positive impact of mobile payment. 

We also constructed the IV Method to overcome the endogenous problem and the PSM method to address the self-selection problems. The results are consistent with baseline regression, implying the robustness of our study.

## 6. Conclusions

The results of this study were as follows: (1) Mobile payment can increase rural household income substantially. (2) While health risks lower the income of rural households, mobile payment can lessen this negative impact. (3) The positive impact of mobile payment is partially explained by the fact that mobile payment can ease liquidity constraints, increase social interaction, and stimulate entrepreneurship.

This paper still has some limitations. (1) We used the cross-sectional data of the CHFS 2017 for our research, which does not consider the period of 2018–2022. Future studies may be supplemented by using panel data. (2) Due to the data availability, we are unable to match the COVID-19 data with household data to explore whether mobile payment can mitigate the negative impact of COVID-19. (3) There are a lot of missing variables when examining the link between mobile payment and rural household income, such as cultural considerations or personality attributes. If these variables can be controlled, the results will be more convincing. To demonstrate the reliability of the baseline results, more techniques should be applied. (4) Additional potential mechanisms need to be discovered to develop a better mobile payment system.

The above findings have the following policy implications. (1) Governments are urged to promote the usage of mobile payment. Possible solutions include enhancing mobile communication networks, implementing smartphone price subsidies, and promoting fundamental knowledge of mobile payments. (2) The feature of mobile payment has to be developed further. Services provided by mobile payment should be expanded and more attention should be paid to the vulnerable groups of rural households. (3) Governments should make great efforts to protect mobile payment users’ rights and control the potential financial risks. (4) Human health is crucial to the development of society and the economy, especially in the background of the global outbreak of COVID-19. The construction of rural medical infrastructure and the medical insurance system should be improved to provide rural households with better medical resources. (5) Rural households’ risk-coping abilities should be enhanced. Possible measures include enhancing their awareness of risk management and prevention, and broadening their channels to obtain aid information.

## Figures and Tables

**Table 1:** Definition of variables.

Variable	Definition
**Dependent Variable**	
Income (RMB)	Ln (household annual total income)
Income_p (RMB)	Ln (household annual total income/the total number of family members)
**Core Independent Variable**	
Mobile payment	=1 if the household uses mobile terminal payment when shopping
Health risks	Number of unhealthy family members
**Other Independent Variable**	
Age	The age of the head of the household
Male	=1 if the head of the household is male
Married	=1 if the head of the household is married
Edu years	Years of education of the head of the household
Work	=1 if the head of the household is employed or self-employed
Asset (RMB)	Ln (household asset)
Household size	Household size measured by the total number of family members
Labor num	Number of family members between 16 and 60 years old
Entrepreneurship	=1 if the household is engaged in business
Per GDP (RMB)	Ln (GDP per capita)
**Mechanism Variable**	
Bank loan	=1 if the household has bank loan
Credit card	=1 if the household has credit card
Transfer expenditure (RMB)	Ln (Transfer expenditure)
Communication cost (RMB)	Ln (Communication cost)
Formal entrepreneurship	=1 if the household is engaged in formal business
Informal entrepreneurship	=1 if the household is engaged in informal business

**Table 2:** Summary Statistics: Key Variables.

Variable	Obs	Mean	Std. Dev.	Min	Max
Income	12,318	9.96	1.50	0.26	17.22
Income_p	12,318	8.68	1.42	0.05	16.52
Mobile payment	12,318	0.11	0.31	0.00	1.00
Age	12,318	57.02	12.27	18.00	97.00
Male	12,318	0.89	0.31	0.00	1.00
Edu years	12,318	7.01	3.45	0.00	19.00
Married	12,318	0.87	0.33	0.00	1.00
Work	12,318	0.74	0.44	0.00	1.00
Household size	12,318	4.12	2.06	1.00	17.00
Labor num	12,318	2.09	1.53	0.00	12.00
Asset	12,318	11.64	1.76	0.69	18.43
Entrepreneurship	12,318	0.10	0.30	0.00	1.00
Per gdp	12,318	10.84	0.34	10.23	11.68
Bank loan	12,318	0.13	0.34	0.00	1.00
Credit card	12,318	0.08	0.27	0.00	1.00
Transfer expenditure	12,318	5.06	3.68	0.00	12.21
Communication cost	12,318	6.74	1.63	0.00	11.00
Formal entrepreneurship	12,318	0.01	0.09	0.00	1.00
Informal entrepreneurship	12,318	0.14	0.34	0.00	1.00

**Table 3:** Summary Statistics: Household Income.

The Use of Mobile Payment	Income	Income_p
YES	11.00	9.48
NO	9.83	8.58

**Table 4:** The impact of mobile payment on rural household income: the OLS Model.

	(1)	(2)	(3)	(4)
	Income	Income	Income_p	Income_p
Mobile payment	1.118 *** (0.038)	0.306 *** (0.036)	0.832 *** (0.038)	0.305 *** (0.036)
Age		0.004 *** (0.001)		0.007 *** (0.001)
Male		−0.009 (0.042)		−0.023 (0.043)
Married		0.040 *** (0.004)		0.040 *** (0.004)
Edu years		0.151 *** (0.039)		0.007 (0.039)
Work		0.208 *** (0.029)		0.196 *** (0.029)
Asset		0.026 *** (0.007)		−0.197 *** (0.007)
Household size		0.272 *** (0.011)		0.248 *** (0.011)
Labor num		0.252 *** (0.008)		0.244 *** (0.008)
Entrepreneurship		0.298 *** (0.038)		0.314 *** (0.038)
Per GDP		0.396 *** (0.139)		0.389 *** (0.139)
Province	Yes	Yes	Yes	Yes
Observations	12,318	12,318	12,318	12,318
Adj. R2	0.078	0.347	0.081	0.276

Notes: The significance levels of 1% is denoted by ***. Robust standard errors are reported in parentheses and province fixed effects are controlled.

**Table 5:** The impact of mobile payment on rural household income: the IV Model.

	(1)	(2)	(3)
	Mobile Payment	Income	Income_p
Mobile payment		2.383 *** (0.348)	2.410 *** (0.346)
Mobile payment usage rate	0.382 *** (0.032)		
Controls	Yes	Yes	Yes
Province	Yes	Yes	Yes
Observations	12,311	12,311	12,311
Adj. R^2^	0.151	0.190	0.095
F Value	40.51		
DWH Test		50.543	53.146

Notes: The significance levels of 1% is denoted by ***. Robust standard errors are reported in parentheses and province fixed effects are controlled. All regressions include the same control variables as in Table 4.

**Table 6:** The impact of mobile payment on household income: the Propensity Score Matching model.

Matching Method	Dependent	Income	Income_p
	Variables		
Nearest neighbor matching (*k* = 1)	ATT	0.248	0.250
	T-stat	4.22	4.24
Nearest neighbor matching (*k* = 4)	ATT	0.226	0.230
	T-stat	4.92	4.92
Radius matching (*r* = 0.01)	ATT	0.980	0.780
	T-stat	9.82	7.96

**Table 7:** The impact of health risk on rural household income: the OLS model.

	(1)	(2)
	Income	Income_p
Health risks	−0.117 *** (0.013)	−0.125 *** (0.013)
Controls	Yes	Yes
Province	Yes	Yes
Observations	12,318	12,318
Adj. R^2^	0.078	0.347

Notes: The significance levels of 1% is denoted by ***. Robust standard errors are reported in parentheses and province fixed effects are controlled. All regressions include the same control variables as in Table 4.

**Table 8:** Moderating effect of mobile payment: the OLS model.

	(1)	(2)	(3)	(4)
	Health Risks Group	No Health Risks Group	Health Risks Group	No Health Risks Group
	Income	Income	Income_p	Income_p
Mobile payment	0.293 *** (0.045)	0.244 *** (0.064)	0.290 *** (0.044)	0.248 *** (0.063)
Controls	Yes	Yes	Yes	Yes
Province	Yes	Yes	Yes	Yes
Observations	6311	6007	6311	6007
Adj. R^2^	0.361	0.302	0.294	0.209

Notes: The significance levels of 1% is denoted by ***. Robust standard errors are reported in parentheses and province fixed effects are controlled. All regressions include the same control variables as in Table 4.

**Table 9:** Mechanism analysis: easing liquidity constraints.

	(1) Bank Loan	(2) Credit Card
Mobile payment	0.027 ** (0.012)	0.158 *** (0.013)
Controls	Yes	Yes
Province	Yes	Yes
Observations	12,318	12,318
Adj. R^2^	0.109	0.086

Notes: The significance levels of 1% and 5% are denoted by *** and **. Robust standard errors are reported in parentheses and province fixed effects are controlled. All regressions include the same control variables as in Table 4.

**Table 10:** Mechanism analysis: increasing social interaction.

	(1) Transfer Expenditure	(2) Communication Cost
Mobile payment	0.670 *** (0.101)	0.301 *** (0.027)
Controls	Yes	Yes
Province	Yes	Yes
Observations	12,318	12,318
Adj. R^2^	0.131	0.341

Notes: The significance levels of 1% is denoted by ***. Robust standard errors are reported in parentheses and province fixed effects are controlled. All regressions include the same control variables as in Table 4.

**Table 11:** Mechanism analysis: stimulating entrepreneurship.

	(1)	(2)	(3)
	Entrepreneurship	Formal Entrepreneurship	Informal Entrepreneurship
Mobile payment	0.122 *** (0.013)	0.016 *** (0.005)	0.141 *** (0.014)
Controls	Yes	Yes	Yes
Province	Yes	Yes	Yes
Observations	12,318	12,318	12,318
Adj. R^2^	0.109	0.016	0.105

Notes: The significance levels of 1% is denoted by ***. Robust standard errors are reported in parentheses and province fixed effects are controlled. All regressions include the same control variables as in Table 4.

## Data Availability

Data used in this study are available upon request.
